# Timing-induced illusory percepts of pitch

**DOI:** 10.1038/s41598-026-53525-0

**Published:** 2026-05-21

**Authors:** Jesse K. Pazdera, Olive M. Rinaldi, Laurel J. Trainor

**Affiliations:** 1 Department of Psychology, Neuroscience and Behaviour, McMaster University, Hamilton, ON Canada; 2 McMaster Institute for Music and the Mind, Hamilton, ON Canada; 3 Rotman Research Institute, Baycrest Hospital, Toronto, ON Canada

**Keywords:** Illusion, Perceptual bias, Perceptual integration, Pitch discrimination, Rhythmic timing, Human behaviour, Auditory system

## Abstract

It has long been proposed that the brain integrates pitch and timing cues during auditory perception. If true, the pitch of a sound should influence its perceived timing, and its timing should influence its perceived pitch. Previous research has found that higher-pitched sounds tend to be perceived as faster than lower-pitched sounds, and in the present study we investigated whether sounds that arrive earlier or later than expected are similarly perceived as higher or lower in pitch. In Experiment 1, participants heard isochronous, repeating standard tones followed by a pitch-shifted probe, and indicated if the pitch increased or decreased. We observed a strong biasing effect of the probe’s timing on its perceived pitch, such that later probes were more likely to be perceived as lower than the standard. Correct, bias-conforming responses to mistimed probes were also significantly faster than responses to on-beat probes. In Experiment 2, we used an adaptive difficulty procedure to investigate whether this timing-induced bias strengthens under conditions of low discriminability. We did not find evidence that bias varies with the magnitude of pitch change or with individual differences in pitch sensitivity. In conjunction with past findings of pitch-induced illusory timing changes, our results support the hypothesis that pitch and time are perceptually integrated. We discuss this integration within a Bayesian predictive coding framework, as possibly learned from real-world correlations between pitch and timing that derive from latent properties of sound sources.

## Introduction

It has long been suggested that timing and pitch are integrated in auditory perception, such that changes along one dimension influence perceived changes along the other^1,2^. Most notably, Jones^2–4^ proposed that the brain integrates the pitch, timing, and loudness of auditory signals into trajectories or vectors through a combined representational space, the structure of which reflects the lawful relations between these three dimensions in the external world. In particular, she emphasized a principle of proportionality, in which changes in pitch (and loudness) are constrained in magnitude by the time over which they occur; meanwhile, changes in time can only be defined through reference to events that themselves have pitch and loudness^2^. Because of lawful relations like proportionality, movement along any one auditory dimension constrains and biases expectations for movement along each other dimension. For example, many studies^1,5–10^ have demonstrated a bias to perceive changes in pitch as larger when spaced over longer intervals (often referred to as *tau* effects), and a bias to perceive the temporal interval between two tones with a larger pitch distance as longer in duration (referred to as *kappa* effects). *Tau* and *kappa* effects have both been attributed to an expectation for pitch to change at a constant velocity over time^7,8,11,12^ (cf.^13^), thereby maintaining proportionality.

Although Jones^2^ focused on the proportionality of the magnitudes of changes in pitch and time, more recent evidence also supports a perceptual link in the *directionality* of changes in pitch and timing. Specifically, there appears to be a perceptual association between pitch increases and temporal acceleration. For example, listeners tend to perceive ascending melodies to be faster^14^ and speeding up more^15^ than descending ones, and applying a continuous pitch glide to a melody also induces illusory changes in tempo^16^. This influence of pitch change on perceived timing extends beyond musical stimuli, as well. Illusions of tempo change have been observed in the perceived modulation rate of frequency-modulated^17,18^ and amplitude-modulated^19^ tones, with ascending tones perceived as increasing in modulation rate. In speech, it has also been found that listeners are best at recognizing changes in speaking rate and pitch when both features change in the same direction^20^.

Effects of absolute pitch on perceived timing have also been observed. For example, higher-pitched speech^21^, melodies^14^, and scales^15^ are perceived as faster than lower pitched ones. Additionally, when participants were asked to make early/late judgments about mistimed probes at the end of a rhythmic sequence, Pazdera and Trainor^22^ found that lower-pitched tones were consistently perceived as later than higher ones. Similarly, the perceptual center of a musical note (the moment in time the sound is perceived to occur) tends to be later for lower-pitched notes^23^, and single intervals flanked by at least one low-pitched tone have been found to be overestimated^24,25^. Recent findings from our own lab suggest that there may be an inverted U-shaped relation between absolute pitch and perceived tempo, in which perceived tempo rises with pitch at lower octaves, but reliably slows above A6 (1760 Hz)^26,27^; however, it remains uncertain whether this U-shaped effect originates from the same mechanism as simple higher–faster illusions^27^.

There has been considerably less investigation into whether faster timing also produces higher perceived pitch; however, if we believe that the brain integrates these two dimensions of sound into a shared representational space, then pitch and timing should bidirectionally influence one another in congruent directions^2,20^. Direct evidence is limited, but one collection of studies has observed a biasing effect of tempo changes on perceived pitch changes, such that slowing the tempo of orchestral and band recordings produced perceived decreases in pitch^28–30^. Additional evidence that the directionality of pitch and timing are at least implicitly associated has been found in musical preference and imagery. When asked to adjust melodies to their preferred tempo, people tend to select faster tempos for higher-pitched music^31^. Similarly, higher pitch correlates with faster imagined motion in adults^32^, though not children^33–35^. Auditory Stroop effects have also been found, in which people appear to associate high pitches with the word “fast” and low pitches with the word “slow”^36^. Further study is needed, however, to conclusively support the perceptual integration of pitch and time.

In the present study, we conducted a pair of pitch discrimination experiments to further investigate whether pitch and timing are integrated in auditory perception. Specifically, we tested whether deviations from isochronous timing can induce perceived changes in pitch. In conjunction with previous findings that pitch changes influence perceived timing, such a reverse-influence of timing changes on perceived pitch would support the hypothesis that pitch and timing are perceptually integrated^2,20^. In both experiments, participants listened to an isochronous, repeating standard tone followed by a (potentially) mistimed final tone that was shifted either up or down in pitch. Participants were tasked with determining the direction of the pitch change, and we analyzed whether the timing offset of the probe tone influenced its perceived pitch. We hypothesized that late probe tones would be more likely to be perceived as low-pitched than early ones, given previous evidence for associations between low pitch and slow timing^14^. We also hypothesized that pitch discrimination would be more sensitive for probe tones played on the beat than for mistimed probes, in line with the principles of Dynamic Attending Theory^37,38^ and previous empirical evidence^39–41^. While we have previously reported an abbreviated analysis of sensitivity and bias in Experiment 1 as part of a conference proceedings^22^, the present manuscript serves the following three novel purposes: 1) to provide a complete analysis and interpretation of Experiment 1, 2) to report a second experiment that investigated whether the difficulty of pitch discrimination moderates the impact of timing changes on perceived pitch, and 3) to discuss pitch and timing illusions under a modern predictive coding framework^42–45^.

## Experiment 1

In Experiment 1, we tested whether the timing of a probe tone influences its perceived pitch. Participants listened to six isochronous standard tones and rated whether a final probe tone was higher or lower in pitch than the standard. The final tone could arrive early, on the beat, or late, and we evaluated whether these timing deviations biased participants’ pitch discrimination responses.

### Methods

#### Participants

We collected data for Experiment 1 between March and April 2022 under special COVID-19 safety protocols, as approved by the local research ethics board, including mask requirements for all participants and experimenters. Thirty undergraduate students (9 male, 21 female) from McMaster University participated in the study for course credit. Ages ranged from 18–22 years, with a mean age of 18.6 ($SD=1.1$). Of these participants, we excluded five from analysis for failing to perform above chance, as evaluated by a binomial test. This study was performed in line with the principles of the Declaration of Helsinki, and approval for the present research was granted by the McMaster Research Ethics Board (No. 2609). All participants provided informed consent prior to their participation in our study.

#### Materials

We used Python to create complex tones with a percussive amplitude envelope by summing four sine waves with random phase, including the fundamental frequency and the first three overtones with an amplitude fall-off of 6 dB/octave. The tones were 250 ms in duration and consisted of a 10 ms linear rise, followed by an exponential decay and 10 ms linear fade. We used Audacity’s loudness normalization function, which is based on recommendation ITU-R BS.1770-4^46^, to balance all tones to the same loudness. To ensure precise inter-onset timing, we pre-generated all tone sequences using Python, and played them back as WAV files during the experiment.

#### Apparatus

Participants completed the study on a 2011 iMac, and we presented stimuli at 75 dBA via a pair of HD 201S Sennheiser headphones. We used the JavaScript library jsPsych^47^ to implement stimulus presentation and response collection. Although we conducted the study in person, we used the online platform Pavlovia (https://pavlovia.org) to host the experiment, which participants accessed via Google Chrome. The purpose of hosting the experiment on Pavlovia was to enable flexible switching between online and in-person testing in the event that COVID-19 restrictions changed during data collection. Ultimately, however, all participants completed the study in person. We performed all analyses using a combination of Python (version 3.12) and R (version 4.3).

#### Design

The study followed a 3 probe timing offset ($15\%$ Early, On-Beat, $15\%$ Late) $\times$ 2 octave ($3^\text {rd}$ or $5^\text {th}$) $\times$ 2 pitch shift direction (Up or Down) within-subjects design. Third-octave standard tones were A3 (220 Hz) and fifth-octave standard tones were A5 (880 Hz), with the probe tone shifted by $\pm 7.9$ cents, equating to $\pm 1$ Hz and $\pm 4$ Hz, respectively.

#### Procedure

Participants completed a pitch discrimination task in which they heard six isochronous repetitions of a standard tone (A3 = 220 Hz or A5 = 880 Hz) followed by a final probe tone. The standard tone always played at an interonset interval of 500 ms, and the probe tone played either 425, 500, or 575 ms after the onset of the final repetition of the standard. Following the presentation of the probe, the participant responded via a key press (up or down arrow) whether the probe was higher or lower in pitch than the repeating standard. There was no time limit on their response, and participants were instructed to respond as accurately as possible. The next trial then began 1.5 s post-response.

Trials were administered in four blocks of 60, with each block consisting of 10 repetitions of each of the six combinations of probe timing offset and pitch shift direction, randomly ordered. In order to reduce the difficulty of the task, all trials within a block used standard tones of the same octave, and octave alternated between blocks in an ABAB pattern. The octave of the first block was randomized between participants. Four practice trials preceded the first block, all of which used a standard pitch of A4 (440 Hz), a probe tone that played 500 ms after the final repetition of the standard, and a pitch shift of $\pm 6$ Hz (three times larger in cents than the experimental trials). Feedback was provided on the practice trials only. Participants received self-paced breaks between blocks.

#### Data analysis

Our primary analysis used a signal detection theory approach to evaluate sensitivity and bias in participants’ pitch discrimination. To do so, we marked trials as hits when participants correctly identified a pitch increase, and we marked trials as false alarms when participants misidentified a pitch decrease as an increase. From this scoring, we calculated sensitivity as $d^\prime$ and bias as *C*^48^, while correcting for hit rates and false alarm rates of 0 and 1 using the method proposed by Hautus^49^. Specifically, we added 0.5 to the numerator and 1 to the denominator when calculating all hit rates and false alarm rates. Under this labeling scheme, higher values of *C* indicate greater conservatism about rating the probe tone as higher in pitch than the standard. In other words, positive values of *C* indicate a bias to rate probes as low-pitched, and negative values of *C* indicate a bias to rate probes as high-pitched. For each participant, we calculated $d^\prime$ and *C* separately for each of the six combinations of probe timing offset and octave. We then analyzed these values via a pair of 3 (offset) $\times$ 2 (octave) repeated measures ANOVAs – one for sensitivity and one for bias.

Next, we performed an exploratory analysis to determine whether the biasing effect of probe timing was stronger in individuals with lower sensitivity. Because our planned analysis found an approximately linear effect of timing on bias, we quantified the magnitude of each participant’s bias by fitting a linear regression across the *C* values for their three probe timing conditions, pooling the data from both octaves. The slope of this line indicates the change in bias associated with every $1\%$ delay of the probe tone. A steeper positive slope therefore indicates a stronger overall bias to rate later probe tones as lower in pitch than earlier probe tones, and we refer to this slope as an individual’s *timing-induced bias*. We then pooled the data from all conditions to calculate each participant’s overall $d^\prime$ sensitivity score, and tested the Pearson correlation between overall sensitivity and timing-induced bias.

Finally, we tested whether reaction times differed depending on probe timing offset and pitch shift direction. For this analysis, we included only correct responses, while excluding any response with a reaction time slower than 5 s ($1.6\%$ of correct responses). We then analyzed reaction times via a 3 (offset) $\times$ 2 (pitch shift direction) repeated measures ANOVA.

### Results

#### Sensitivity & bias

**Fig. 1:**
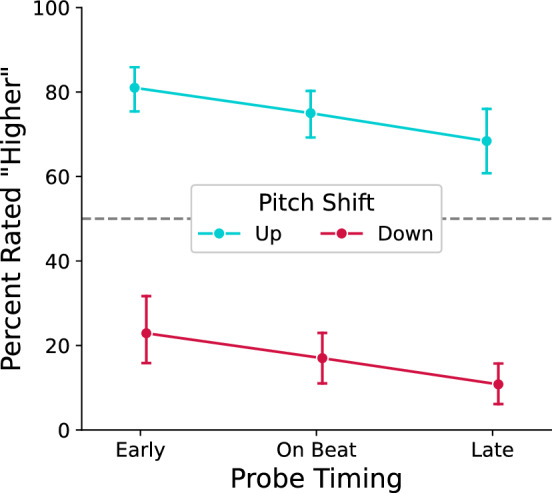
Probability of rating probe tones as higher than the repeating standard tone in Experiment 1, depending on the probe tone’s timing and true pitch shift direction. Error bars indicate within-subject 95% confidence intervals^50^. In calculating $d^\prime$ and *C* for all subsequent analyses, we treated correctly-rated pitch increases as hits (upper line) and incorrectly-rated pitch decreases as false alarms (lower line). Hit rates and false alarm rates both decreased as the probe tone became later.

**Fig. 2:**
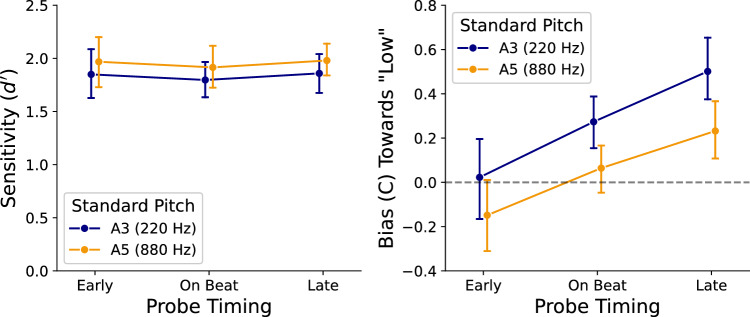
Sensitivity ($d^\prime$) and bias (*C*) of pitch discrimination in Experiment 1, as a function of the octave of the standard tone and the timing offset of the probe tone. Higher values of *C* indicate a greater bias towards labeling probe tones as lower in pitch than the standard. Error bars denote within-subject $95\%$ confidence intervals. The timing of the probe tone biased participants’ pitch perception at both octaves, such that later probes were perceived as lower, without a reduction in discriminability.

**Fig. 3:**
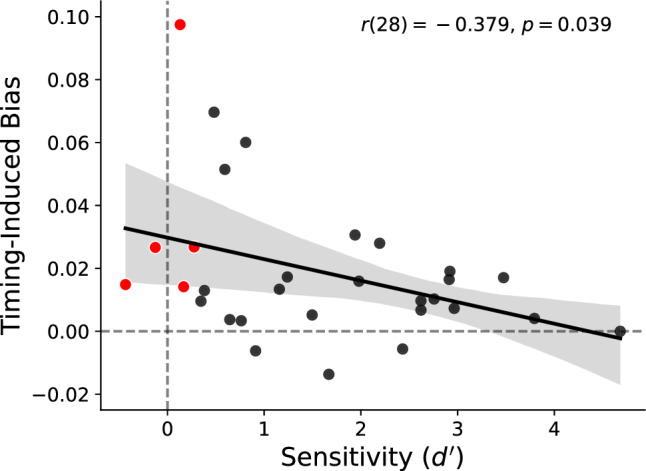
Individual differences in $d^\prime$ and timing-induced bias. Each data point represents one participant’s sensitivity ($d^\prime$) and timing-induced bias scores in Experiment 1. Higher timing-induced bias scores indicate a stronger tendency to rate later tones as lower in pitch than the standard. Participants marked in red are those who were excluded from other analyses for failing to perform above-chance. The shaded region indicates the regression line and its 95% confidence interval. Individuals who were less sensitive to pitch changes tended to be more biased by the probe’s timing offset.

Figure 1 illustrates the percent of probe tones participants rated as higher in pitch than the standard, as a function of probe timing offset and the true pitch shift direction. These data suggest that the earlier the probe tone played, the more likely participants were to rate it as higher in pitch. In order to separately analyze sensitivity and bias within these ratings, we labeled all trials where participants correctly identified pitch increases (upper line) as hits, and labeled all trials where participants incorrectly responded to pitch decreases (lower line) as false alarms. From these hit rates and false alarm rates, we obtained the sensitivity ($d^\prime$) and bias (*C*) of participants’ pitch discrimination. Figure 2 illustrates sensitivity and bias as a function of the octave of the standard tone and the timing offset of the probe tone. Higher values of $d^\prime$ indicate greater discriminability of pitch increases and decreases, while higher values of *C* indicate a bias towards rating probe tones as lower in pitch than the standard.

We analyzed sensitivity via a 3 (offset) $\times$ 2 (octave) repeated measures ANOVA. Neither probe timing offset, $F(2, 48)=0.26$, $p=.772$, $\omega _p^2=-.010$, nor octave, $F(1, 24)=0.74$, $p=.397$, $\omega _p^2=.010$, significantly affected sensitivity, and offset and octave did not interact, $F(2, 48) \approx 0.00$, $p>.999$, $\omega _p^2=-.014$. Participants were similarly sensitive to pitch changes at both octaves, and regardless of whether the probe tone played early, on the beat, or late.

We next analyzed bias via a 3 (offset) $\times$ 2 (octave) repeated measures ANOVA, which indicated significant main effects of both probe timing offset, $F(2, 48)=10.51$, $p<.001$, $\omega _p^2=.290$, and octave, $F(1, 24)=8.99$, $p=.006$, $\omega _p^2=.133$. The interaction between timing offset and octave was not significant, $F(2, 48)=0.42$, $p=.661$, $\omega _p^2=-.008$. Post-hoc pairwise *t*-testing with Holm–Bonferroni correction indicated that the *C* values for all three probe timing offsets significantly differed from one another, with participants tending to rate later probe tones as lower in pitch, as hypothesized. This pattern was consistent between both octaves we tested; however, participants showed an unexpected main effect of octave such that they were more likely to rate probe tones as lower in pitch when the standard was A3 (220 Hz) than when it was A5 (880 Hz).

Finally, we explored whether the biasing effect of the probe’s timing correlated with sensitivity. Figure 3 illustrates each participant’s overall $d^\prime$ across all trials, paired with the magnitude of their bias to rate later probe tones as lower (formally, the linear slope of their bias across offset conditions, see Data Analysis). Participants who failed to perform above chance, and were therefore excluded from our main analyses, are marked in red. Notably, participants with low $d^\prime$ values tended to be highly biased by timing, especially those with $d^\prime <1$. When including all participants, we observed a moderate negative correlation between sensitivity and timing-induced bias, $r(28)=-.379, p=.039$. Among above-chance performers, this correlation remained moderate in size, but was non-significant, $r(23)=-.330, p=.107$.

#### Reaction time

**Fig. 4:**
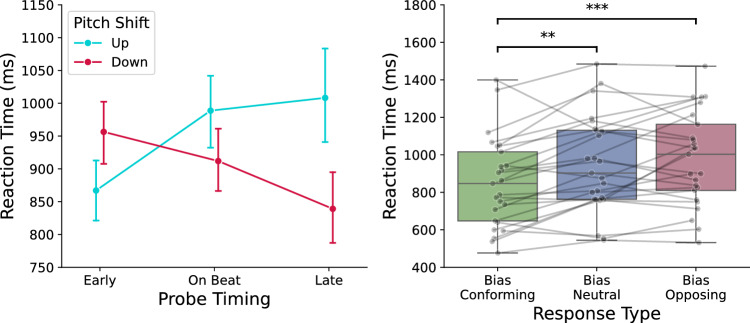
Reaction times for correct responses in Experiment 1. **A** Participants correctly rated pitch increases most quickly when the probe tone was early (blue line), but correctly rated pitch decreases most quickly when the probe was late (pink line). Error bars indicate within-subject 95% confidence intervals. **B** Categorizing responses as either bias-conforming (early/high or late/low), bias-neutral (all responses to on-beat probes), or bias-opposing (early/low or late/high) reveals faster reaction times for bias-conforming responses than for both other response categories. Individual data points indicate subject averages for each category.

Figure 4A illustrates average reaction times for correct pitch discrimination responses, depending on the direction of the pitch shift and the timing offset of the probe tone. A 3 (probe timing offset) $\times$ 2 (pitch shift direction) repeated measures ANOVA identified a significant two-way interaction, $F(2, 48)=13.38$, $p<.001$, $\omega _p^2=.142$, while neither main effect was significant: probe timing offset, $F(2, 48)=1.32$, $p=.276$, $\omega _p^2=.003$, and pitch shift direction, $F(1, 24)=1.78$, $p=.194$, $\omega _p^2=.035$. In particular, Fig. 4A suggests that participants responded correctly most quickly to early, high probes and late, low probes. Therefore, as a post-hoc test of the two-way interaction between timing offset and pitch direction, we recategorized correct responses as either bias-conforming (early/high and late/low probes), bias-opposing (early/low and late/high probes), or bias-neutral (any on-beat probe). We next calculated each participant’s average reaction time when making each of these three response types, as shown in Fig. 4B. We then conducted dependent samples *t*-tests with Holm–Bonferroni correction between each of the three response types. As expected, reaction times for bias-conforming responses ($M=850$ ms, $SD=241$ ms) were significantly faster than reaction times for both bias-neutral ($M=949$ ms, $SD=255$ ms), $t(24)=-3.88$, $p_{adj}=.001$, and bias-opposing responses ($M=980$ ms, $SD=250$ ms), $t(24)=-4.42$, $p_{adj}<.001$. However, bias-opposing responses were not significantly slower than bias-neutral responses, $t(24)=1.24$, $p_{adj}=.233$.

### Discussion

In a pitch discrimination paradigm, we observed a biasing effect of a probe tone’s timing on the perception of its pitch. As hypothesized, when a pitch-shifted probe tone played 15% early following six isochronous repetitions of a standard, participants showed a bias to rate the probe as higher in pitch than the standard; meanwhile, when the probe played 15% late, participants showed a bias to rate it as lower in pitch (Fig. 2). Our results support the idea that pitch and timing are integrated during auditory perception^2^. In conjunction with previous findings that higher pitches and ascending pitch sequences are perceived as faster^14^, earlier^22^, or speeding up^17^, our results support a bidirectional influence in which timing can also influence perceived pitch.

To better understand the biasing effect of tone timing on pitch perception, we also analyzed participants’ reaction times. We found that correct, bias-conforming responses to early and late probes (i.e., early/high and late/low) were approximately 100 ms faster on average than correct judgments of on-beat probes. In contrast, bias-opposing responses (i.e., early/low and late/high) were not significantly slower than responses to probe tones that played on the beat. We provide a detailed interpretation of this pattern of reaction times in the General Discussion.

The timing-induced bias we identified was not accompanied by a decrease in sensitivity to pitch changes; indeed sensitivity was quite consistent across early, on-beat, and late probe tones, in contrast with our hypothesis that $d^\prime$ would be highest for on-beat probes. One possible explanation for the lack of a dynamic attending-style advantage for on-beat perception^37–41^ is that an on-beat sensitivity advantage might require a design in which the majority of probes fall on the beat. In the current design, the probe only played at the “expected” time on one third of trials. Although the average probe timing was on the beat, participants may have learned to spread their attention across the full presentation window due to the high variability in the probe’s timing^38^. Alternatively, as each trial was only about 3 s in length, trials may have been too short for dynamic attending to emerge. For comparison, previous dynamic attending advantages for pitch perception were found in sequences lasting 50 s^39^.

In addition to time biasing perceived pitch change, participants also unexpectedly showed a bias to rate probe tones as lower than the standard when the standard was A3 (220 Hz), but they were relatively unbiased on average when the standard was A5 (880 Hz; see Fig. 2). It is possible that our results relate to the pitch class polarization phenomenon identified by Prpic and colleagues^51^, in which musicians tended to underestimate the pitch class of lower-octave tones and overestimate the pitch of higher-octave tones. However, given substantial differences between our pitch discrimination task and their pitch class identification task, further investigation would be necessary to support a definitive link between our findings.

Lastly, we identified a possible negative correlation between sensitivity and timing-induced bias, such that participants with low sensitivity tended to be more strongly biased by the probe tone’s timing (Fig. 3), but only when we included participants who failed to perform above-chance in the analysis. We designed Experiment 2 to investigate two potential explanations for such a correlation. One possibility is that people may rely on temporal cues as supplemental information when they are uncertain about a pitch change. In this case, we should be able to observe a within-subject effect of task difficulty on timing-induced bias. By varying the size of the pitch shift between trials in Experiment 2, we tested whether individuals would increasingly rely on timing information as pitch changes diminished. Alternatively, individuals with greater pitch sensitivity may simultaneously be better able to differentiate pitch changes from timing changes, allowing them to resist the bias. In Experiment 2, we measured participants’ just-noticeable differences for pitch change, and used this measure to calibrate the task difficulty on an individual basis. If greater pitch sensitivity is associated with improved separability of pitch and timing information, then participants with smaller just-noticeable differences should also tend to show weaker timing-induced bias in Experiment 2.

## Experiment 2

In Experiment 2, we followed up on our exploratory finding that individuals with lower sensitivity to pitch change also tended to show stronger timing-related bias. To do so, we created an adaptive-difficulty version of our pitch discrimination task. We first determined each participant’s $70.7\%$ just-noticeable pitch difference (JND) via a staircase procedure in which they rated which of two tones was higher in pitch. After obtaining their JND, we presented them with a task similar to Experiment 1, except that the probe tone shifted by a number of cents either equal to their JND (easier condition), or half that number (harder condition). If timing-related bias is stronger in individuals with weaker pitch sensitivity, then we would expect the effect of probe timing offset to positively correlate with JND (as higher JNDs indicate lower sensitivity). Alternatively, or in addition, if timing-related bias increases with task difficulty, then we would expect a stronger effect of probe timing offset in the harder pitch shift condition than in the easier condition.

### Methods

#### Participants

We collected data for Experiment 2 between February and April 2023, under the same COVID-19 safety protocols as Experiment 1. Twenty-eight undergraduate students (17 female, 11 male) from McMaster University participated for course credit. Ages ranged from 18-22 years ($M=18.8$, $SD=1.2$). We excluded one participant from analysis for failing to perform above chance, as determined via a binomial test. An additional 13 (12 female, 1 male) undergraduate students aged 18-20 years ($M=18.4$, $SD=0.6$) completed an alternative version of the task in which all trials were presented at their JND, and these participants were included only in our analysis of whether JND predicts timing-induced bias. Approval for this research was granted by the McMaster Research Ethics Board (No. 2609), and all participants provided informed consent before the experiment.

#### Materials

Tones were constructed via the same procedure as Experiment 1, with the exception that loudness normalization across octaves was not required due to all tones being within 100 cents of A4 (440 Hz).

#### Apparatus

Participants completed the study on a Windows 10 computer with an Asus Z87-C motherboard, and we presented stimuli at 78 dBA via a set of Escape HP-3868 headphones. We implemented stimulus presentation in Python (version 3.8) using the PsychoPy library^52^, and performed all analyses using Python (version 3.12) and R (version 4.3).

#### Design

The main pitch discrimination task followed a 3 probe timing offset ($15\%$ Early, On-Beat, or $15\%$ Late) $\times$ 2 difficulty (Easy or Hard) $\times$ 2 pitch shift direction (Up or Down) fully within-subjects design. The easier difficulty condition used pitch changes equal to the participant’s JND, whereas the harder difficulty condition used pitch changes equal to one half the participant’s JND. Pilot testing suggested that with practice, participants became quite good at differentiating pitch changes at their JND, and we found that setting the more difficult condition to be below their initial JND produced desirable levels of performance.

#### Procedure

The session began with a difficulty calibration task, in which we determined the participant’s 70.7% just-noticeable difference for pitch discrimination via an interleaved staircase procedure. On each trial of the calibration task, participants heard a 440 Hz tone followed by a tone slightly higher or lower in pitch than the first, with a 500 ms interonset interval between them. Participants then answered via a key press (1 or 2) whether the first or second tone was higher. A 1.5 s delay followed their response before the next trial began. We used four interleaved staircases in a 2 pitch direction (first tone higher or second tone higher) $\times$ 2 initial pitch shift size (1 cent or 25 cents) design. On each trial, we selected one staircase at random to generate the stimuli for that trial. We used a two-down, one-up procedure such that two consecutive correct answers on trials generated by the same staircase increased the difficulty of the next trial generated by that staircase, reducing the number of cents by which the tones differed (to a minimum of 0); meanwhile, a single incorrect answer reduced the difficulty of the next trial generated by that staircase, increasing the number of cents by which the tones differed (to a maximum of 100). Initially, difficulty changed by 8 cents at a time, and this step size halved after every two reversals in difficulty on a per-staircase basis, to a minimum step size of 1 cent. Each staircase ended after eight reversals in difficulty. After all four staircases had ended, we calculated the participant’s JND as the average pitch shift size of the last four reversals from each staircase.

We next used the JND obtained from the calibration task to generate probe tones that were a number of cents above and below the standard tone (A4) equal to that threshold, as well as probe tones that were above and below the standard tone by one half the JND. Participants then completed a pitch discrimination task that followed the same procedure as Experiment 1, with the exception that the standard tones were always A4 and the size of the pitch difference between the standard and probe (JND or $\frac{1}{2}$JND) varied across trials. Trials were again organized into four blocks of 60 separated by breaks, with each combination of probe timing offset, difficulty (pitch shift size), and pitch shift direction presented five times per block in a fully randomized order. Four practice trials with a pitch shift size of four times the JND preceded the first block. Feedback was given on the practice trials only.

#### Data analysis

We calculated participants’ sensitivity and bias in each condition in the form of $d^\prime$ and *C*, respectively, using the same methods as Experiment 1^48,49^. To confirm that our difficulty manipulation affected sensitivity as intended, we first analyzed $d^\prime$ via a 2 (difficulty) $\times$ 3 (probe timing offset) repeated measures ANOVA. Next, to assess whether difficulty affected the strength of the later–lower timing bias on pitch perception, we quantified timing-induced bias in a similar manner to Experiment 1. Specifically, for each participant and each difficulty, we fit linear models across the *C* values for the three probe timing offset conditions. As before, the slope of this line quantifies the expected change in bias with each $1\%$ delay in the timing of the probe tone, which we refer to as the *timing-induced bias*. We then compared the timing-induced bias values from the two shift size conditions using a paired-samples *t*-test. To determine whether individuals with more sensitive pitch perception were less biased by timing, we calculated the Pearson correlation between participants’ JNDs and their timing-induced bias, specifically for trials presented at their JND (the Easy condition).

### Results

#### Sensitivity & bias

**Fig. 5:**
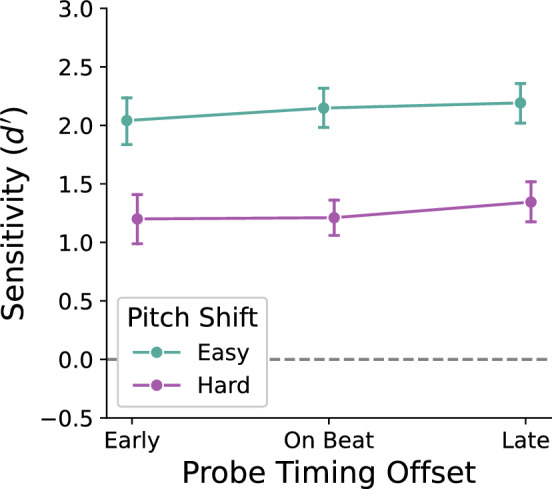
Pitch discriminability by difficulty level. Lines indicate pitch discrimination performance in Experiment 2, based on the size of the pitch shift (Easy = JND; Hard = $\frac{1}{2}$JND) and the timing of the probe tone. Error bars indicate within-subject 95% confidence intervals. Increasing the difficulty of the task by reducing the size of the pitch shift successfully reduced $d^\prime$ evenly across probe timing conditions.

**Fig. 6:**
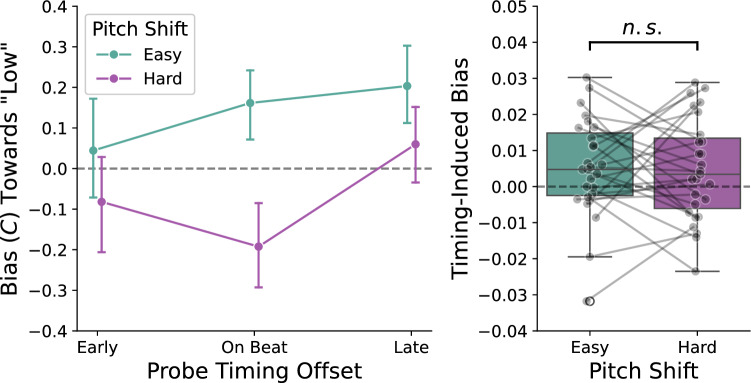
Bias in pitch discrimination as a function of the probe tone’s timing and the size of the pitch shift in Experiment 2. **A** Average bias (*C*) towards rating probe tones as lower than the standard in each condition. Error bars indicate within-subject 95% confidence intervals. Late tones were more likely to be rated as low-pitched than early and on-beat tones. **B** Data points indicate the linear effect of probe timing offset on bias for each participant and each difficulty condition. Reducing the size of the pitch shift did not strengthen timing-induced bias.

We first assessed whether our difficulty manipulation produced lower levels of sensitivity on trials where the pitch shift size was $\frac{1}{2}$JND than on trials where it was equal to their JND. Figure 5 illustrates $d^\prime$ for each combination of difficulty and probe timing offset. A 2 (difficulty) x 3 (probe timing offset) repeated measures ANOVA identified a large, significant main effect of difficulty on $d^\prime$, $F(1, 26)=49.97$, $p<.001$, $\omega _p^2=.469$, a non-significant main effect of of probe timing offset, $F(2, 52)=1.59$, $p=.214$, $\omega _p^2=.005$, and a non-significant interaction, $F(2, 52)=0.18$, $p=.836$, $\omega _p^2=-.010$. Smaller pitch shifts were significantly less discriminable than larger pitch shifts, confirming that our difficulty manipulation was successful. Furthermore, consistent with Experiment 1, participants were similarly sensitive to pitch changes regardless of whether the probe played early, late, or on the beat.

Having confirmed that our difficulty manipulation impacted pitch discriminability, we next tested whether difficulty affected participants’ tendency to rate later probes as lower. Figure 6A illustrates *C* as a function of difficulty and the probe’s timing offset. A 2 (difficulty) x 3 (probe timing offset) repeated measures ANOVA identified significant main effects of difficulty, $F(1, 26)=9.88$, $p=.004$, $\omega _p^2=.152$, and probe timing offset, $F(2, 52)=4.67$, $p=.014$, $\omega _p^2=.067$, as well as a significant two-way interaction, $F(2, 52)=3.72$, $p=.031$, $\omega _p^2=.032$. The main effect of difficulty was such that participants showed an overall bias to rate larger pitch shifts as a decrease and smaller pitch shifts as an increase. With respect to the effect of the probe’s timing, post-hoc pairwise *t*-tests with Holm–Bonferroni correction found that late probes were rated as significantly lower than early and on-beat probes. To determine whether the two-way interaction matched our hypothesis that timing-induced bias would be stronger when the pitch shift was smaller, we compared timing-induced bias between pitch shift sizes (Fig. 6B) using a dependent samples *t*-test. According to our hypothesis, timing induced bias should be more positive in the JND condition than the $\frac{1}{2}$JND condition; however, this was not the case, $t(26)=0.18$, $p=.860$. Rather, the two-way interaction can be accounted for by the difference in *C* being significantly larger between difficulty conditions when the probe played on the beat than when it played late, $t(26)=2.57$, $p=.016$.

#### Just-noticeable differences & timing-induced bias

**Fig. 7:**
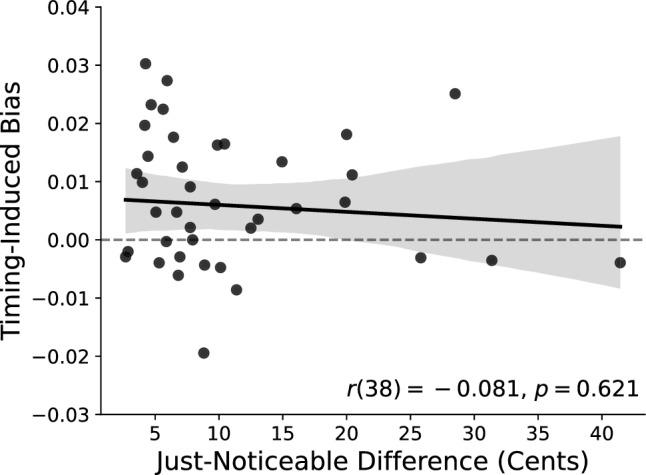
Individual differences in JND and timing-induced bias. Data points indicate each participant’s just-noticeable pitch difference in cents, paired with their timing-induced bias in Experiment 2. Higher just-noticeable differences indicate less sensitive pitch perception, while greater timing-induced bias indicates a stronger tendency to rate later tones as lower. The shaded region indicates the regression line and its 95% confidence interval. Participants in Experiment 2 were similarly biased by the probe tone’s timing regardless of their pitch sensitivity.

Figure 7 illustrates each participant’s $70.7\%$ just-noticeable pitch difference alongside the timing-induced bias they exhibited on pitch discrimination trials presented at their JND. A positive correlation would indicate that the biasing effects of probe timing were stronger among participants with less sensitive pitch perception (consistent with Fig. 3 from Experiment 1), after accounting for task difficulty. Instead, we observed a weak and non-significant negative correlation, $r(38)=-.081$, $p=.621$, suggesting that timing biased participants’ pitch perception similarly regardless of their sensitivity to pitch differences.

### Discussion

In Experiment 2 we investigated whether the biasing effects of timing on pitch perception vary in strength according to task difficulty and/or individual pitch sensitivity. We conducted an adaptive-difficulty pitch discrimination task calibrated to each person’s just noticeable pitch difference. Although we replicated the bias to perceive later probe tones as lower in pitch, we did not find evidence that this bias strengthens when pitch changes are made less discriminable by reducing the size of the change. Timing-induced bias was similarly strong when the pitch change was equal to the participant’s JND as when it was half that size (Fig. 6). We also did not find evidence that the strength of the bias correlated with JND (Fig. 7). Participants were similarly influenced by the timing of the probe regardless of the precision of their pitch perception. Therefore, neither of these factors appear to account for the sensitivity–bias correlation in Experiment 1.

## General discussion

Across two pitch discrimination experiments we observed a biasing effect of early versus late tone timing on perceived pitch. Later timing resulted in lower perceived pitch without an impact on discriminability (Figs. 1, 2, 5, 6). The strength of this illusion was not found to depend on task difficulty, operationalized as the magnitude of the pitch difference between standard and probe tones (Fig. 6), nor was it found to correlate with individual differences in sensitivity to pitch changes (Fig. 7). Alongside previous findings that ascending pitch produces illusions of speeding up^14,15,17^, our results provide evidence that pitch and timing bidirectionally influence one another in auditory perception. This bidirectional influence is consistent with accounts that suggest pitch and timing are perceptually integrated^2,20^.

This type of cue integration has often been framed as a Bayesian inference problem^53–58^, and we can apply a similar explanation here. The fundamental idea behind Bayesian models of perception is that the brain needs to infer the state of the surrounding environment based on noisy sensory information, in conjunction with learned priors regarding the statistical structure of the world. Due to the stochastic nature of neural activity, there are many different states of the world that can produce any given pattern of sensory activation; therefore, incorporating prior knowledge and beliefs about world structure helps narrow down which of these possible world states generated any given pattern of sensory activity. These principles have coalesced into the modern predictive coding theory of the brain^42–45^, including predictive coding accounts of music perception and auditory processing^59–62^.

Under a predictive coding framework, the function of auditory perception is to identify objects in the world and track their movement^63^. The periodicity of an auditory signal like a musical rhythm or the repeating tones from the present study can provide evidence about the movement of the signal’s source. A simple example is that one can infer how fast a person is moving by listening to the intervals between their footsteps. A shortening of the intervals suggests they are speeding up, whereas a lengthening of the intervals suggests they are slowing down. Likewise, in our stimuli, the shortening or lengthening of the final interval suggests that the signal’s latent source-object is speeding up or slowing down, respectively. Recent Bayesian models of rhythm perception dynamically track tempo by a similar process, continuously updating inferences about the phase and period of a latent source that generates observable musical notes^59,64,65^. In this way, sensory observations continuously inform an ongoing inference about the state and trajectory of their latent sources.

When multiple sensory cues are inferred to come from the same causal source – especially if they derive from a single property of that source (e.g., size, location, or velocity) – the brain is believed to integrate the cues via a hierarchical network in order to optimally infer the state of the source^54^. Each cue updates the estimates of the source-object’s properties, and the current estimates of the source-object’s properties bias the perception of each cue towards what would be expected if those were the true properties. This integration has previously been used to explain illusions in perceived visual^66^ and tactile^67^ motion, and may also account for the effects observed in the present study.

Returning to our example of speeding up or slowing down, the inferred velocity of an object should simultaneously influence expectations for all sensory properties that tend to co-vary with velocity. The most commonly cited explanation for pitch-induced illusions of tempo change is a real-world correlation between higher pitches and faster timing, either due to both features co-varying with arousal / energetic state in biological sound sources^14,15,21,24^ or due to the physical mechanics of sound production in objects like engines^14^ and musical instruments^68^. For example, in music, lower-pitched instruments tend to be larger and have slower attack times, constraining how quickly they can be played relative to smaller, higher-pitched instruments. If the pitch and timing of an auditory signal are both cues to some of the same properties of a sound source (e.g., movement velocity, energetic state, size), then bidirectional pitch–timing illusions should arise as a natural consequence of a predictive coding or Bayesian integration process. Changes in either the pitch or timing of the signal update the inferred state of the source, and changes in the inferred state of the source inform expectations for both the pitch and timing of the sounds it emits.

As it relates to our study, specifically, the tones occurring at regular intervals imply a certain movement velocity or energetic state for their sound source. When the final tone arrives earlier or later than expected, it signals that the source has increased or decreased in energy, respectively. And as long as pitch is also a cue to changes in energy as hypothesized above, this inferred change in energy will bias perceived pitch. The same principle may explain why pitch changes have also been observed to produce illusory tempo changes^14–19^, and a similar process may underlie effects of intensity on perceived pitch^69–71^ and timing^14,21^, as faster and more energetic objects may tend to make louder sounds. This dynamic resembles Jones’^2–4^ hypothesis that perceived pitch, timing, and loudness should influence one another based on their real-world correlations, but it differs in that this influence is mediated through the inference of a hidden variable such as the energy or movement of the sound source, rather than being directly determined by correlations between auditory dimensions.

Other pitch and timing illusions, such as auditory *tau* and *kappa* effects, may derive from other properties of object motion, such as a prior expectation for constant velocity^7,8,11,12^ (cf.^13^). Under this prior, the inferred trajectory of an object is a mix of the pitch and timing that was sensed and the pitch and timing that should have been observed if the object moved with constant velocity^12^. The consequence is that more time is perceived to have elapsed the greater pitch has changed^6,7,10^, and pitch is perceived to have changed more when a larger interval has elapsed^1,5,8^, similar to Jones’^2^ concept of proportionality of movement along auditory dimensions.

### Faster bias-conforming responses

In addition to identifying a bias to perceive early tones as high-pitched and late tones as low-pitched, we found that correct, bias-conforming responses to early and late probes were around 100 ms faster on average than correct responses to on-beat probes. Correct bias-opposing responses were also slightly slower on average than correct responses to on-beat probes, but not significantly so (Fig. 4). As this analysis was inherently correlational, we cannot conclude whether timing-induced bias allowed for faster responding or whether faster responses produced more biased answers. One possibility is that congruent pitch and timing cues produce faster responses due to faster evidence accumulation in a decision process relative to when an incongruent cue is present^72^. Similar explanations have been proposed to explain interference and facilitation effects in the classic Stroop paradigm^73^.

Alternatively, faster pitch judgments may tend to be more biased by timing. In this case, bias-conforming responses may be more likely when the participant responds quickly, which might also result in bias-conforming responses having the shortest mean reaction time. In optimal cue integration, the weighting of each cue depends on its precision^74,75^. If timing deviations are processed faster than deviations in the frequency spectrum of the stimulus, the precision of timing cues would likely reach a maximum earlier than the precision of spectral cues in informing estimates of the tone’s pitch. If this were the case, timing should initially bias pitch perception strongly, then decline in influence as the spectral cue becomes more reliable. A similar pattern of results would be expected from an evidence accumulation model in which timing cues become available earlier than spectral cues^73^. To test the hypothesis that timing-induced bias declines over the response interval, future research might experimentally manipulate reaction times by introducing response deadlines that require participants to respond within a limited time window. Requiring participants to give faster responses would be predicted to increase timing-induced bias, if this hypothesis were correct.

### Difficulty and perceptual sensitivity

**Fig. 8:**
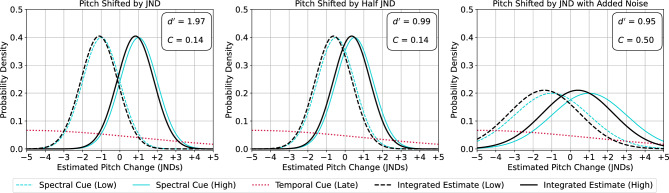
Example of Bayesian cue integration for a late probe tone that is either lower (dashed lines) or higher (solid lines) in pitch than the standard. Turquoise curves illustrate idealized, unbiased estimates of pitch change based only on the frequency spectrum of the probe tone. The dotted red curve implements a prior expectation $(\mathcal {N} (\mu =-5, \sigma =6))$ for a late tone to be lower than the standard. Black curves illustrate pitch estimates based on the precision-weighted combination of both the spectral and temporal cues, and the provided $d^\prime$ and *C* values indicate the sensitivity and bias of these integrated estimates under three difficulty conditions. Bias remains constant regardless of whether pitch is shifted by the participant’s JND (left panel) or one half their JND (center panel), corresponding to our Easy and Hard conditions in Experiment 2, respectively. In contrast, degrading the spectral cue (right panel) would be expected to increase timing-induced bias.

Precision-weighted cue integration may also explain why we did not find evidence for an effect of task difficulty on timing-induced bias in Experiment 2. If we consider timing-induced bias to be the result of a Bayesian cue integration process between spectral and temporal cues to a tone’s pitch, then the strength of this bias would depend primarily on the relative reliability of these cues for identifying the tone’s pitch.

Figure 8 illustrates this principle by showing how two distinct forms of difficulty manipulation should differentially affect timing-induced bias under a Bayesian framework. The left panel illustrates cue integration in a task similar to our Easy condition in Experiment 2, where each participant heard probe tones that were shifted up (solid lines) or down (dashed lines) in pitch by their individual JND. The frequency spectrum of a tone provides an unbiased cue for estimating its pitch, represented by the two probability distributions outlined in turquoise. The tone arriving later than expected introduces a bias (outlined in red) to perceive the tone as lower in pitch than the standard. As a result, the integrated pitch estimates (black curves) – computed by multiplying the turquoise and red distributions together – are more negative than the pitch estimates based on the tone’s frequency spectrum alone. The center panel illustrates our Hard difficulty condition, in which we reduced the pitch shift to one half the participant’s JND. As we observed in our study, this manipulation would be expected to approximately halve $d^\prime$ without increasing timing-induced bias. In precision-weighted cue integration, $\textit{C}$ remains constant between these two difficulty conditions because the tone’s spectral information is equally reliable in both conditions – the spectrum (and its neural encoding) just differs less between “high” and “low” probes. In contrast, the right panel shows that increasing difficulty by degrading the frequency spectrum of the probe tone (e.g., through spectral smearing^76^) would be expected to increase timing-induced bias alongside reducing $d^\prime$. Future research should therefore investigate whether degrading pitch clarity increases timing-induced bias, where reducing discriminability at a fixed level of sensory precision did not. Such a pattern of results would further support the hypothesis that illusory pitch derives from a Bayesian cue integration process.

Given a precision-weighted integration process, we might also expect individuals with less precise pitch perception to down-weight spectral information and up-weight temporal information. In Experiment 1, we did observe a correlation between lower $d^\prime$ and stronger timing-induced bias that conforms to this prediction (Fig. 3), but only when including participants who failed to perform above chance on the pitch-discrimination task. In Experiment 2, we did not observe a similar correlation between bias strength and just-noticeable differences for pitch change (Fig. 7). Thus, we found little evidence overall that temporal cues influence perceived pitch more strongly in people with relatively poor pitch sensitivity. One possibility is that individuals with poor pitch sensitivity also tend to have reduced temporal sensitivity^77^, in which case the relative reliability of spectral and temporal cues may generally be stable across individuals. Future studies of timing-induced illusory pitch should consider measuring participants’ JNDs for both pitch and timing to help address this question.

As for why individuals with low $d^\prime$ did show a relatively strong bias in Experiment 1, one possibility is that some participants explicitly resorted to using timing as a decision heuristic when frequently unable to detect pitch changes. In situations where participants can only detect one feature of a stimulus changing, they may conceivably resort to making judgments based on that feature, even if it is not the feature to which they were instructed to attend^21^. Calibrating pitch changes individually for each participant in Experiment 2 may have eliminated the need for this compensatory strategy. Thus, timing-induced bias in pitch discrimination may originate both from an automatic sensory bias that is always active, alongside an additional decision bias that arises only when making judgments about pitch changes too small to detect. The latter is unlikely to happen outside of laboratory settings, but is an important consideration for future research design.

### Rhythmic deviation or foreperiod effect?

Although we used deviations from a rhythmic context to manipulate perceived pitch in the present study, it is possible that timing-induced bias depends on the time elapsed since the end (or beginning) of the previous note, rather than on a note’s phase within its rhythmic context. This alternative explanation could be tested using a pitch discrimination task in which a single standard tone plays on each trial, followed by a variable delay (i.e., foreperiod) before the onset of the probe tone. At least two studies^78,79^ have implemented a design similar to this without testing the effects of foreperiod duration on bias, as reaction time and accuracy have typically been the focus of foreperiod analyses. We believe our present results support the addition of tests for foreperiod effects on bias in future studies of pitch discrimination. If shorter foreperiods in the absence of a rhythmic context result in higher perceived pitch, our present results might be better explained not by a learned association between changes in pitch and timing, but rather by an effect in which residual neural activity from one tone exerts a decaying pitch bias on the perception of the next. However, given our previous findings that pitch influences perceived mistiming in the same direction that mistiming influences perceived pitch^22^, we believe that a learned correlation is more likely.

## Conclusion

The present study demonstrates that a tone’s timing within a rhythmic context can alter the perception of its pitch. Specifically, tones that arrive earlier than expected are perceived as higher in pitch, while those that arrive later than expected are perceived as lower in pitch. These timing-induced illusory pitch changes – alongside previous evidence for pitch-induced illusory tempo changes – support the long-standing hypothesis that the brain integrates pitch and timing during auditory perception.

## Data Availability

We have made all data, code, and stimuli from both experiments publicly available on the Open Science Framework at https://osf.io/hrj3t/, as well as on GitHub at https://github.com/jpazdera/IllusoryPitch.
